# Antidepressant drug use after intensive care: a nationwide cohort study

**DOI:** 10.1038/s41598-024-66028-7

**Published:** 2024-07-09

**Authors:** Erik von Oelreich, Jesper Eriksson, Mikael Eriksson, Emma Larsson, Anders Oldner

**Affiliations:** 1 Perioperative Medicine and Intensive Care, Karolinska University Hospital, Solna, 171 76 Stockholm, Sweden; 2 Section of Anaesthesiology and Intensive Care Medicine, Department of Physiology and Pharmacology, Karolinska Institutet, Stockholm, Sweden; 3 Department of Anaesthesia and Intensive Care, Uppsala University Hospital, Uppsala, Sweden; 4 Department of Surgical Sciences, Anaesthesia and Intensive Care, Uppsala University, Uppsala, Sweden

**Keywords:** Psychotropic drugs, Antidepressant agents, Behavioural symptoms, Depression, Suicide, Epidemiology, Pharmacoepidemiology, Epidemiology, Risk factors

## Abstract

Modern intensive care has improved survival rates, but emerging evidence suggests a high prevalence of post-intensive care unit (ICU) health problems, including post-traumatic stress disorder, depression and anxiety. These symptoms may have a detrimental effect on quality of life and increase mortality. The primary objective of this study is to examine the extent of initiation of antidepressant medication among ICU survivors and identify the factors associated with its usage. The secondary objective is to investigate whether the use of these medications is linked to an increased mortality. The nationwide study cohort included 125,130 ICU survivors admitted between 2010 and 2017. Within the first 3 months after ICU discharge, 7% of patients initiated antidepressant medication, by 1 year 15.5% had started medication. We found no tendency to a decrease during the 2-year follow-up period. Factors associated with antidepressant use included middle age, female sex, psychiatric and somatic comorbid conditions, substance dependence, higher illness severity, and longer ICU stay. Antidepressant users had a higher mortality rate, and deaths due to external causes and suicide were more frequent in this group. This study emphasizes the importance of detecting and addressing depression in ICU survivors to improve their quality of life and reduce mortality rates.

## Introduction

Modern intensive care has proven its success by saving more lives compared to the past. However, emerging data shed light on health issues that arise after the intensive care unit (ICU) episode. Several reports suggest that a considerable proportion of patients discharged from ICUs face not only somatic disorders but also significant psychiatric health challenges including post-traumatic stress disorder, depression and anxiety. This cluster of symptoms is commonly referred to as post-intensive care syndrome^1,2^. Despite its apparent prevalence, the extent of this problem remains largely unexplored on a broader scale.

Critically ill patients commonly face significant psychological challenges in the ICU setting. Within an environment of limited communication and reduced autonomy, patients frequently endure pain, delirium, and potentially associated psychotic experiences. Sleep disturbances, nightmares, and intrusive memories are commonly experienced. The triggers related to their illness can evoke intense emotions and vivid mental images, which may manifest physically or emotionally, contributing to depression after discharge from the ICU.

It has been estimated that almost a third of ICU survivors experience clinically significant depressive symptoms within the first year following critical illness^3^. The presence of these symptoms can restrict patients' involvement in physical activities and hinder their social participation, consequently impeding rehabilitation, and recovery from critical illness. It is widely recognized that depression is associated with increased mortality and morbidity, particularly among individuals with chronic medical conditions^4^.

Primary objective of this study was to describe the magnitude of initiation of antidepressant medication in a nation-wide cohort of ICU survivors. Secondary objectives included identifying risk factors for SSRI use and the association between use of antidepressant medication after ICU discharge and increased risk of death.

## Methods

### Ethics Approval

This study was approved by the regional ethical review board in Stockholm, Sweden (approval numbers 2018/2541-31 and 2019-00213) and waived the requirement for informed consent. All research was performed in accordance with national guidelines and regulations.

### Study Design

The study cohort includes all entries in the Swedish Intensive Care Registry (SIR) between 2010 and 2017. SIR is a national quality register for intensive care and collects information from ICUs in Sweden including data on demography, procedures, and mortality. Admissions to the cardio-thoracic ICU care were excluded, as most of these patients are admitted for postoperative care after cardiac surgery. Patients using antidepressants prior to admission were also excluded, as we aimed to study the initiation of antidepressant medication following ICU care. Patients dying during the first 3 months after admission were excluded since we were primarily interested in investigating long-term outcomes. If patients had more than one care episode registered, the first one was included. Estimated mortality rate (EMR) was calculated from the APACHE (acute physiology and chronic health evaluation) score for admissions 2010–2012^5^. For later entries the EMR was calculated from SAPS (simplified acute physiology score)^6^. Mortality risk estimates based on acute physiology scores such as the Simplified Acute Physiology Score (SAPS) and the Acute Physiologic Assessment and Chronic Health Evaluation (APACHE) are utilized in clinical practice to evaluate disease severity, used in outcome research and when evaluating the performance of an ICU. These scores are based on the worst data obtained within the first 24 h post-admission. Data on comorbidities was collected from the Swedish National Patient Register^7^ up to 5 years before admission. Level of education at the time of ICU admission was defined as low, medium or high corresponding to ≤ 9 years (primary school), 10–12 years (secondary school) and > 12 years (postsecondary school) respectively. Level of income was divided into low, medium, and high corresponding to 0.5 times the median income as low, 0.5–2 times the median income as medium, and > 2 times the median income as high income. The information on education and income was retrieved from the Longitudinal Integration Database for Health Insurance and Labour Market Studies (LISA) managed by Statistics Sweden^8^. Detailed information on mortality was assessed in The Swedish Cause of Death Register^9^ and information on prescribed drugs in The Swedish Prescribed Drug Register^10^. The Swedish Prescribed Drug Register, overseen by the Swedish Board of Health and Welfare (NBHW), encompasses data on every prescribed medication dispensed within the country. Since 1 July 2005, it has incorporated personal identity numbers, facilitating its integration with other databases from that point forward. The registry only contains information about drugs that are prescribed and collected outside of the hospital. Thus, there is no information in the registry about the drugs used while a patient is hospitalized. The register is considered to have 100 per cent coverage, encapsulating all medications necessitating a prescription. In the context of Sweden, antidepressants are exclusively available through prescriptions and can only be procured at community pharmacies.”

### Outcomes

Primary outcome was prescribed and dispensed antidepressant medications during the first year following critical care and the secondary outcome death 12–18 months following critical care.

### Definition of antidepressant use

Use of antidepressants before critical care equalled at least one written and dispensed prescription (Anatomical Therapeutic Chemical (ATC) Classification System codes starting with N06A) during 6 months preceding ICU admission. Use of antidepressant medication after ICU care was defined as at least one dispensed prescriptions of the same ATC-codes in the first 12 months following ICU care. In Sweden a prescription covers 3 months and therefore quarter periods were used.

### Statistical analysis

Multivariable logistic regression models were performed to calculate odds ratios (ORs) for associations between use of antidepressant use and clinically relevant risk factors (age, sex, income, education level, psychiatric and somatic comorbid conditions, substance dependence, EMR, surgical intervention and critical care length of stay).

Cox proportional hazards model were used to assess a potential association between antidepressant medication and mortality after critical care admission. The results were presented with hazard ratios (HR). Known or potential confounders (age, sex, psychiatric anc somatic comorbid conditions, substance dependence, EMR, critical care length of stay) were selected before the study. Follow-up time in the Cox regression analysis was 12–18 months after ICU admission. The proportional-hazards assumption was tested on the basis of Schoenfeld residuals.

### Sensitivity analyses

To assess non-random dropout due to death, probability weights were used in the multivariable logistic regression model^11^. The probability of dying within the first 3 months following admission to critical care was estimated with a logistic regression model including all covariates in the multivariable model and with the addition of the year of critical care admission. Separate analyses of the multivariable logistic regression model and the Cox regression model were performed excluding patients with prior psychiatric diagnosis.

### Missing data

Due to missing data on education, income and EMR, three separate analyses were conducted, each excluding one of these variables.

*P* values < 0.05 were deemed to be statistically significant. Stata/SE 16.1 was used for computer analyses. Further, the study complied with the Strengthening the Reporting of Observational Studies in Epidemiology (STROBE) recommendations for cohort studies^12^.

## Results

**Figure 1:**
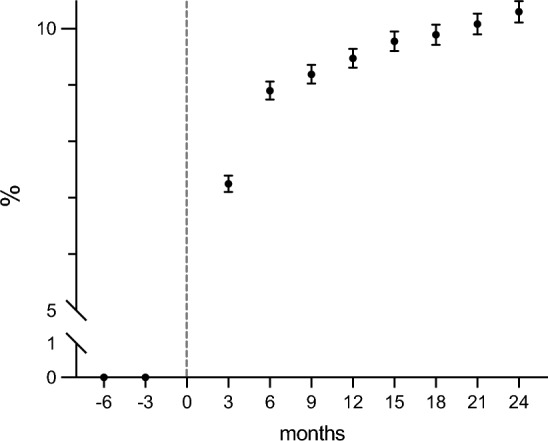
Dispensed prescriptions of antidepressants in relation to ICU care. Proportion of patients obtaining dispensed prescriptions of antidepressants each 3-month period (quarter) before and after ICU admission. Dotted line depicts the time of ICU admission. Median proportion with IQR depicted by black circles.

From 2010 to 2017, 237,904 patients were collected in SIR. Following exclusion of 31,358 patients admitted to thoracic intensive care, 45,783 patients dying in the first 3 months after critical care admission, and 35,633 using ant-depressant drugs 6 months prior to ICU admission, 125,130 patients were included in the final study cohort (Additional File 1, Figs. S1). Table 1 includes characteristics of the study cohort. Seven percent of ICU survivors without previous use of antidepressant drugs, received prescriptions of antidepressants within the first 3 months after discharge (Fig. 1). At 1 year after ICU admission 19,347 (15.5%) patients had started antidepressant medication (Table 1). There was no decrease in the frequency of dispensed prescriptions throughout the 24-month follow-up period (Fig. 1). Patients with de novo antidepressant medication (n = 19,347) were younger, more likely to be female, had a higher prevalence of psychiatric comorbidity, and more commonly a history of substance abuse upon ICU admission. Additionally, antidepressant users had a longer length of stay in the ICU as well as higher EMR when admitted to intensive care (Table 1).

**Table 1:** General characteristics in included ICU patients stratified by use of antidepressant medication during the first 12 months after ICU care.

	Non-SSRI user	SSRI user	Missing data (%)
Count	105,783 (84.5)	19,347 (15.5)	
Age	63 (44, 73)	60 (43, 72)	
Male	64 104 (60.6)	10,787 (55.8)	
Income categories			764 (0.61)
Low	19,523 (18.6)	3670 (19.1)	
Medium	80,599 (76.7)	14,824 (77.0)	
High	5002 (4.8)	748 (3.9)	
Education categories			3550 (2.83)
Low	37,155 (36.2)	6665 (35.2)	
Medium	45,288 (44.1)	8712 (46.0)	
High	20,179 (19.7)	3581 (18.9)	
CCI categories			
CCI 0	50,751 (48.0)	9205 (47.6)	
CCI 1	17,910 (16.9)	3525 (18.2)	
CCI > 1	37,122 (35.1)	6617 (34.2)	
History of psychiatric illness	11,081 (10.5)	4296 (22.2)	
History of substance abuse	10,250 (9.7)	3111 (16.1)	
Acute myocardial infarction	8580 (8.1)	1512 (7.8)	
Congestive heart failure	10,154 (9.6)	1686 (8.7)	
Cerebrovascular disease	8750 (8.3)	2289 (11.8)	
COPD	11,285 (10.7)	2214 (11.4)	
Rheumatoid disease	3272 (3.1)	647 (3.3)	
Liver disease	6300 (6.0)	1252 (6.5)	
Diabetes	20,782 (19.6)	3789 (19.6)	
Renal disease	4965 (4.7)	841 (4.3)	
Cancer	19,594 (18.5)	3347 (17.3)	
EMR, median (IQR)	0.065 (0.018, 0.18)	0.073 (0.024, 0.19)	919 (0.73)
ICU length of stay, days			
0–2	65,953 (62.3)	11 026 (57.0)	
3–7	30,728 (29.0)	5220 (27.0)	
> 7	9102 (8.6)	3101 (16.0)	
Surgery			
Acute care	13,243 (12.5)	2318 (12.0)	
Elective	11,448 (10.8)	1631 (8.4)	
No surgery	81,092 (76.7)	15,398 (79.6)	

Categorical parameters are presented as n (%), continuous parameters as median with interquartile range (IQR), CCI, Charlson Comorbidity Index; COPD, Chronic Obstructive Pulmonary Disease; EMR, Estimated Mortality Rate; ICU, Intensive Care Unit.

**Figure 2:**
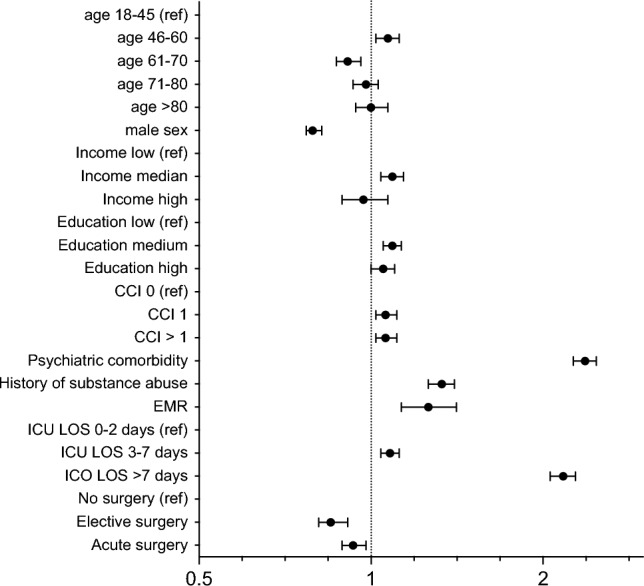
Factors associated with initiation of antidepressants. Multivariable logistic regression analysis of potential factors associated with initiation of antidepressant medication. Logarithmic scale for odds ratios with 95% confidence intervals on the x-axis. CCI Charlson’s Comorbidity Index. EMR Estimated Mortality Ratio, ICU LOS Intensive Care Unit Length of Stay.

In the multivariable logistic regression analysis: female sex, being middle aged, a higher level of education, somatic and psychiatric comorbidity, a history of substance abuse, higher EMR and ICU stay for more than two days as well as absence of surgery were associated with higher odds of antidepressant use (Fig. 2, Table S1).

**Figure 3:**
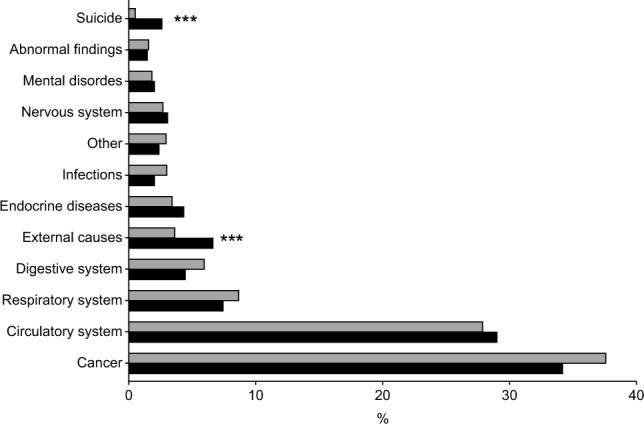
Causes of death for ICU patients using antidepressant compared with non-users. Causes of death for patients surviving the first year after ICU admission, but who died during months 12 to 18 post ICU-care (n = 4206). Grey bars depict non-antidepressant users (*n* 3335), black bars depict antidepressant users (*n* = 871). ***p < 0.001 by Chi square test.

During the follow up period after critical care admission, 4206 patients died, of whom 871 were new antidepressant users. In the unadjusted Cox proportional hazards analysis, initiation of antidepressant medication was associated with a higher mortality, HR 1.5 (1.4–1.6 95% CI *P* < 0.001). After adjusting for age, sex, psychiatric and somatic comorbid conditions, substance dependence, EMR and length of stay in the ICU; the association remained significant, HR 1.6 (1.4–1.7) 95% CI, *P* < 0.001). The assumption of the proportional hazards in the Cox regression model indicated no evidence of non-proportional hazards. When analysing causes of death, individuals with antidepressant drug use after ICU care displayed significantly more deaths due to external causes and suicide (Fig. 3).

In the analysis assessing non-random dropout due to death being middle aged, EMR, absence of acute surgery and length of ICU stay 3–7 days were no longer significantly associated with antidepressant use after ICU care.

When excluding patients with a prior psychiatric diagnosis the results were unchanged both for factors associated with initiation of antidepressant medication and for the risk of death.

### Missing data

Smaller numbers of missing data were found on education (n = 3550, 2.83%), income (n = 764, 0.61%) and EMR (919, 0.73%) (Table 1). The proportion of missing data in education, income and EMR is less than 5%, which has been suggested as the maximum upper acceptance limit for large datasets^13^. Moreover, the distribution of the response variables of interest was very similar in the analyses comparing observations with/without missing education, income and EMR, indicating a non-informative missingness mechanism^14^.

## Discussion

In this nationwide cohort study, over 15% of ICU survivors without previous use of antidepressant drugs, started medication during the first year after ICU care. Moreover, there was no signal of a decrease in dispensed prescriptions throughout the 24-month follow-up period. Factors associated with use of antidepressant medication included being middle aged, being female, psychiatric and somatic comorbid conditions, substance dependence, high EMR and an length of ICU stay for more than 2 days. Mortality rates between 6 and 18 months after ICU care were higher for individuals who had initiated antidepressant medication.

With advances in critical care medicine, an increasing number of survivors are discharged from ICUSs^15^. This notion has prompted a growing emphasis on long-term outcomes, encompassing mental health and cognitive results. In this context a high prevalence of depressive symptoms has been observed among ICU survivors. A systematic review reported that the prevalence of clinician-diagnosed depressive disorders was as high as 33% across studies, considerably higher than the general 1-year prevalence among US adults^3,16^. Another study found that up to 6 months following long-term mechanical ventilation in the ICU, 20% of chronically critically ill patients were affected by major depression^17^.

In our study, we found that more than 15% of ICU survivors initiated new antidepressant medication within the first year. This percentage should be compared to an anticipated initiation rate. According to a large study conducted in the UK, the initiation rate of antidepressants was reported to be 2.15 per 100 person-years^18^. This indicates that the rate in our cohort was over seven times higher, highlighting the significant impact of post-critical illness depression. Moreover, we noted no trend to a reduction in the use of antidepressants over the two-year follow up time. A study conducted in Denmark found that compared to a matched cohort of hospitalised patients and to the general population, ICU survivors had an increased use of psychotropic medications, particularly antidepressants and sedative-hypnotics^19^. Interestingly, and in contrast to the results of the current study, these differences had largely resolved by 9 to 12 months after discharge.

Several factors linked to the initiation of antidepressant medication following ICU care were identified, including being female and being in the middle age range. Several previous studies have associated female sex and younger age with depressive symptoms after ICU care^20–22^. However, two recent systematic reviews reported that neither young age nor sex were consistently associated with depressive symptoms after ICU care^3,19^. A high level of education was associated with use of antidepressants similar to a U.S report where antidepressant medication use was higher for adults with college education compared with those with a high school education^23^. In contrast, low level of education was associated with depressive symptoms in a US cohort of ICU survivors^21^. The initiation of antidepressant medication was associated with somatic comorbidity and a history of psychiatric illness, which aligns with existing evidence indicating that patients with physical disorders are at a higher risk of experiencing depression. Moreover, prior psychopathology has been linked to both depressive symptoms and post-traumatic stress disorder following ICU care^24–27^. A history of substance abuse increased the odds of initiating antidepressant medication, in line with studies showing that nearly one-third of patients with major depressive disorders also have a history of substance use^28^. The finding that higher EMR was associated with antidepressant use contradicts several reviews that demonstrate the severity of critical illness is not a consistent predictor of depressive symptoms and diagnosis.^3,27,29^. The length of ICU stay, to some extent a proxy for the severity of illness, was also associated with medication initiation in our study. This association has been reported previously^26^, while other studies have found no association between the length of stay and depressive symptoms at follow-up^29^. Overall, some of the risk factors found in the current study are not consistently reported in the literature.

The initiation of new antidepressant medication was found to be associated with an elevated risk of death 12–18 months after ICU admission. Additionally, individuals who used antidepressants experienced a higher incidence of deaths attributed to external causes and suicide compared to non-users. Previous studies have shown critical care followed by a depressive disorder is associated with increased mortality for up to 2 years after discharge [30, 31]. Furthermore, a recent study also found that survivors of critical illness had increased risk of suicide and self-harm consistent with our findings^32^.

Several studies presenting data on symptoms of depression following exposure to critical illness report incidences in range of 25–36%^16–18^. As our study cohort was limited to patients starting antidepressant medication these figures suggest that the number of patients experiencing depressive symptoms in our cohort might be substantially higher than the 15.5% subject to medication.

In the ICU, several factors such as age, sex, reason for admission, illness severity, use of sedation and length of stay have not consistently been associated with the development of depressive symptoms post-discharge^17^. The risk factors for initiation of antidepressant medication presented in our study are similar to some previous studies but differ from others. Therefore, screening for post-ICU depression should be comprehensive and include individuals of all ages and sexes, as well as those with different disease severity and length of stay. Focusing only on patients with severe illness or certain characteristics may result in overlooking a large number of symptomatic patients^4^. Although a systematic review did not find strong evidence supporting the use of post-ICU interventions for treating depressive symptoms^29^, studies have indicated that post-ICU outpatient physical rehabilitation interventions may reduce depressive symptoms^33^. Clearly, further research is warranted to explore the potential benefits of rehabilitation and exercise interventions for ICU survivors^34^.

## Strengths and limitations

One strength of the study is the fact that all registered ICU entries in Sweden are included. Furthermore, the national health registries used in the study are validated with low rates of missingness. The study being retrospective and register-based is a limitation. The generalizability might be limited by different healthcare system in different countries. The study's focus on de novo antidepressant medication does not fully encompass the issue of post-critical illness depression. Furthermore, we only studied prescribed and dispensed medication, and cannot be sure to what extent the individuals were taking their medication or not. In addition, we have no data on the in-hospital quantities of antidepressants given to the patients. In Table S1 there is a potential Table 2 fallacy where we cannot know whether for a specific factor-outcome association, all the variables in the model are potential confounders or mediators^35,36^. However, in this study we do not have any real primary exposure since all our patients have de facto been exposed to intensive care and we have no control group that remains unexposed. The best would have been to evaluate the exposure of intensive care for a group of patients and then have a matched control group, but such data extraction was not possible. Therefore, we present all potential confounders even though one must be very careful not to draw any far-reaching conclusions when interpreting the data.

## Conclusions

Our results demonstrate that initiation of de novo antidepressant medication was substantial for ICU survivors, over 15% started medication within the first year after ICU admission and initiation of antidepressant medication was associated with increased mortality. Detecting and recognising depression is crucial as it can lead to a decreased quality of life and is associated with higher mortality rates. Post-critical illness depression is a significant condition with the potential for treatment and prevention, demanding enhanced attention and care for ICU survivors.

### Supplementary Information

Supplementary Information 1.

Supplementary Information 2.

## Data Availability

The data that support the findings of this study are available from The Swedish Intensive Care Registry and national health registers. Restrictions apply to the availability of these data, which were used under license for the current study, and so are not publicly available. Data is however available from the authors upon reasonable request and with permission of The Swedish Intensive Care Registry and the Swedish National Board of Health and Welfare.

## Abbreviations

ICU: Intensive care unit
SIR: Swedish Intensive Care Registry
EMR: Estimated mortality rate
APACHE: Acute physiology and chronic health evaluation
SAPS: Simplified acute physiology score
ATC: Anatomical therapeutic chemical
LISA: Longitudinal integration database for health insurance and labour market studies
NBHW: Swedish Board of Health and Welfare
SSRI: Selective serotonin reuptake inhibitors
HR: Hazard ratio
OR: Odds ratio
