# Supplementary figures and images for: Antidepressant drug use after intensive care: a nationwide cohort study
Source: Sci Rep. 2024 Jul 9;14:15863. doi: 10.1038/s41598-024-66028-7 (PMC11233594; doi:10.1038/s41598-024-66028-7)

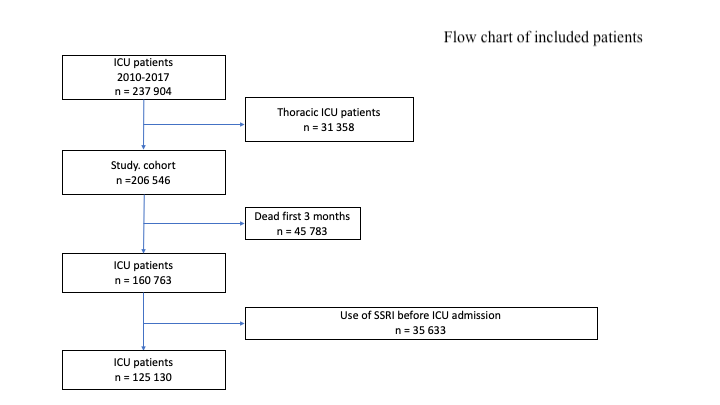

Supplement: Supplementary file 1 — Supplementary Information 1. [file 41598_2024_66028_MOESM1_ESM.tiff]
