# Supplementary material for: Antidepressant drug use after intensive care: a nationwide cohort study
Source: Sci Rep. 2024 Jul 9;14:15863. doi: 10.1038/s41598-024-66028-7 (PMC11233594; doi:10.1038/s41598-024-66028-7)
Supplement: Supplementary file 2 — Supplementary Information 2. [file 41598_2024_66028_MOESM2_ESM.docx]

**Table S1.** Univariate and multivariable logistic regression analyses, associations with initiation of antidepressant medication presented as OR (95% CI).

|  | **Univariate** | **P value** | **Multivariable** | **P value** |
| --- | --- | --- | --- | --- |
| **Age categories**  **18-45**  **46-60**  **61-70**  **71-80**  **80-** | Ref.  1.08 (1.03-1.12)  0.85 (0.81-0.86)  0.86 (0.83-0.90)  0.85 (0.80-0.90) | 0.001  < 0.001  < 0.001  < 0.001 | Ref.  1.07 (1.02-1.12)  0.91 (0.87-0.96)  0.98 (0.93-1.03)  1.00 (0.94-1.07) | 0.005  0.001  0.44  0.90 |
| **Male** | 0.82 (0.79-0.85) | < 0.001 | 0.79 (0.77-0.82) | < 0.001 |
| **Income categories**  **Low**  **Medium**  **High** | Ref.  0.98 (0.94-1.02)  0.80 (0.73-0.87) | 0.28  < 0.001 | Ref.  1.09 (1.04-1.14)  0.97 (0.89-1.07) | < 0.001  0.57 |
| **Education level**  **Low**  **Medium**  **High** | Ref.  1.07 (1.04-1.11)  0.99 (0.95-1.03) | < 0.001  0.63 | Ref.  1.09 (1.05-1.13)  1.05 (1.00-1.10) | < 0.001  0.038 |
| **CCI categories**  **CCI 0**  **CCI 1**  **CCI > 1** | Ref.  1.09 (1.04-1.13)  0.98 (0.95-1.02) | < 0.001  0.32 | Ref.  1.06 (1.02-1.11)  1.06 (1.02-1.11) | 0.008  0.003 |
| **Psychiatric comorbidity** | 2.44 (2.35-2.54) | < 0.001 | 2.37 (2.26-2.48) | < 0.001 |
| **Substance abuse** | 1.79 (1.71-1.87) | < 0.001 | 1.33 (1.26-1.40) | < 0.001 |
| **EMR** | 1.49 (1.36-1.63) | < 0.001 | 1.26 (1.13-1.41) | < 0.001 |
| **ICU length of stay, days**  **0-2**  **3-7**  **> 7** | Ref.  1.02 (0.98-1.05)  2.04 (1.95-2.13) | 0.38  < 0.001 | Ref.  1.08 (1.04-1.12)  2.17 (2.06-2.28) | < 0.001  < 0.001 |
| **Surgery**  **No surgery**  **Elective**  **Acute care** | Ref.  0.75 (0.71-0.79)  0.92 (0.88-0.97) | < 0.001  0.001 | Ref.  0.85 (0.81-0.91)  0.93 (0.89-0.98) | < 0.001  0.007 |

CCI, Charlson Comorbidity Index; ICU, Intensive Care Unit; EMR, Estimated Mortality Rate.
